# The Elongation Factor 1 Alpha Promoter Drives the Functional Expression of Kir2A in *Plutella xylostella* Cells

**DOI:** 10.3390/ijms26073042

**Published:** 2025-03-26

**Authors:** Yinna Wang, Haihao Ma, Zheming Liu, Piao Zhao, Jia Liu, Hang Zhu, Yong Zhou, Yilong Man, Xiaomao Zhou

**Affiliations:** 1 Longping Branch, College of Biology, Hunan University, Changsha 410125, China; wangyinna@hnu.edu.cn; 2 College of Forestry, Central South University of Forestry and Technology, Changsha 410004, China; 3 Institute of Plant Protection, Hunan Academy of Agricultural Sciences, Changsha 410125, China; liuzheming2007@hunaas.cn (Z.L.); zhaopiao@hunnu.edu.cn (P.Z.); jialiuv@hunaas.cn (J.L.); zhumstrong@hunaas.cn (H.Z.); qmbchg@hunaas.cn (Y.Z.); yilongman@hunaas.cn (Y.M.); 4 Hunan Provincial Key Laboratory of Pesticide Biology and Precise Use Technology, Changsha 410125, China; 5 Key Laboratory of Pesticide Assessment, Ministry of Agriculture and Rural Affairs, Changsha 410125, China

**Keywords:** *Plutella xylostella*, EF1α promoter, Kir2 channel, cell line

## Abstract

Cell lines and their corresponding expression plasmids are extensively utilized in the study of insect physiology and pathology. In this research, four single-cell cultured lines (Px4-1 to Px4-4) of *Plutella xylostella* were established from eggs. The promoter for the *P. xylostella elongation factor 1α* (PxEF1α), known for its high driving activity in cells, was cloned and used to construct expression plasmids. Dual-luciferase activity assays and EGFP expression analyses demonstrated that the PxEF1α promoter exhibited the strongest driving activity in Px4-2 cells, comparable to that of the immediate-early 1 promoter associated with the homologous region 5 enhancer (AcIE1^hr5^) from the *Autographa californica* multicapsid nucleopolyhedrovirus (AcMNPV). In contrast, the driving activity of PxEF1α in cells derived from *Spodoptera frugiperda*, *Trichoplusia ni*, and *Helicoverpa armigera* was lower. Furthermore, the PxEF1α promoter was successfully employed to drive inward rectifier potassium 2A (Kir2A) expression in Px4-2 cells. The electrophysiological properties of the insect Kir2A channel were successfully characterized for the first time. It was observed that the PxKir2A channel possesses typical inward rectifier potassium channel properties and can be inhibited by nanomolar concentrations of VU625 and VU590. This study offers a novel approach for the expression and investigation of foreign gene function in insect cells and provides a valuable tool for the in-depth study of key biomolecules in *P. xylostella*.

## 1. Introduction

Insect cell lines and their culture techniques have been extensively utilized in research areas such as insect physiology, pathology, and molecular biology [1]. These cell lines, which originate from Lepidopteran insects—including *Spodoptera frugiperda* [2], *Helicoverpa armigera* [3], *Spodoptera litura* [4], *Trichoplusia ni* [5], and *Bombyx mori* [6]—play a crucial role in biological research and production applications. They are commonly employed in studies of the infection mechanisms of insect baculoviruses, microsporidia, and other obligate parasites [7,8]. Additionally, these cell lines facilitate the large-scale production of recombinant viruses for use in pesticides [9] and the expression of important proteins with either research or commercial value [10]. Primary culture and subculture techniques using egg or pupa tissue are the main methods for obtaining insect cell lines [11]. Currently, a significant number of insect cell lines continue to be established and reported each year [12].

The promoter plays a critical role in directing, regulating, and targeting transgene expression [13]. Increased promoter activity can lead to the administration of lower doses of vectors, resulting in reduced toxicity and fewer side effects [13]. Obtaining promoters that can effectively initiate the expression of target genes in insect cells is essential for elucidating protein heterologous expression, biomolecular functions, cellular signaling pathways, and protein interactions [14]. The immediate-early 2 promoter from the *Orgyia pseudotsugata* multicapsid nucleopolyhedrosis virus (OpIE2), along with the immediate-early 1 (IE1) promoter from the *Autographa californica* multicapsid nucleopolyhedrovirus (AcMNPV), which is associated with the homologous region 5 enhancer (AcIE1^hr5^), is commonly used for the expression of foreign genes in insect cells [15,16]. However, when the same promoter is repeatedly used to drive multiple foreign genes, gene silencing or co-repression may occur, further limiting its application in transgenic insect research [17]. Additionally, promoters derived from insect constitutive genes, such as actin and ubiquitin, are also widely utilized in insect cell expression [18,19]. However, substantial evidence indicates that the same promoter exhibits significant variability in activity across different insect cell types [20]. Currently, the selection of promoters in the study of non-model organisms continues to face considerable challenges [20].

Inwardly rectifying potassium (K^+^) channels, known as Kir channels, facilitate the preferential movement of K^+^ ions into the cell rather than out of it [21]. These channels play a crucial role in regulating cellular excitability and maintaining K^+^ ion homeostasis [22]. In mammals, Kir channels are essential for regulating heartbeat, renal function, cellular excitability, and insulin release [23]. Three subclasses of Kir channel subunits (Kir1-3) have been reported in insects [21]. Currently, Kir1 channels from various insect species, such as *Drosophila melanogaster* [24], *Aedes aegypti* [25], *Anopheles gambiae* [26], *Nilaparvata lugens* [27], *Aphis glycines* [28], and *Apis mellifera* [29], can be expressed in HEK293T cells or *Xenopus* oocytes, allowing for the recording of channel currents using voltage clamp techniques. Additionally, Kir1 channels have been found to influence important physiological activities in insects, including feeding, Malpighian tubule excretion, silk gland secretion, and oviposition [21]. Research studies on the electrophysiological and biological functions of Kir2B channels in insects, such as *A. aegypti*, have also been published [30]. Kir2A and Kir2B belong to the Kir2 subfamily [31]; however, there are currently few reports on the electrophysiological characteristics and biological functions of these channels, which limits our understanding of Kir2 channels. Interestingly, studies involving *D. melanogaster* Kir2 have shown that wild-type DmKir2 was unable to detect channel currents using voltage clamp techniques when expressed in HEK293T cells and *Xenopus* oocytes [24]. In contrast, channel currents were successfully detected when expressed in *D. melanogaster* S2 cells, providing a reference for the study of other insect Kir2A channels [24].

In a previous study, we attempted to express the Kir2A channel in HEK293T cells and record the channel current using single-cell patch clamp technology; however, this effort was unsuccessful. We also expressed Kir2A in *Xenopus* oocytes but were unable to record the channel current in that system as well. To achieve a more natural state for the *P. xylostella* Kir2A channel, we first constructed a cell line derived from *P. xylostella* embryos. The transcriptome analysis revealed that the *elongation factor 1 alpha* (*PxEF1α*) gene was highly expressed in these cells. Subsequently, we cloned and characterized the PxEF1α promoter, and we compared the transcriptional activities of four promoters across four different lepidopteran cell lines. Ultimately, we successfully expressed the Kir2A channel in *P. xylostella* cell lines using the PxEF1α promoter, and we assessed the electrophysiological characteristics of PxKir2A using single-cell patch clamp technology. This study provides valuable data for investigating the gene expression and protein function in insect cell lines and serves as a reference for the electrophysiological study of insect ion channels.

## 2. Results

### 2.1. Establishment of P. xylostella Cell Lines

**Figure 1:**
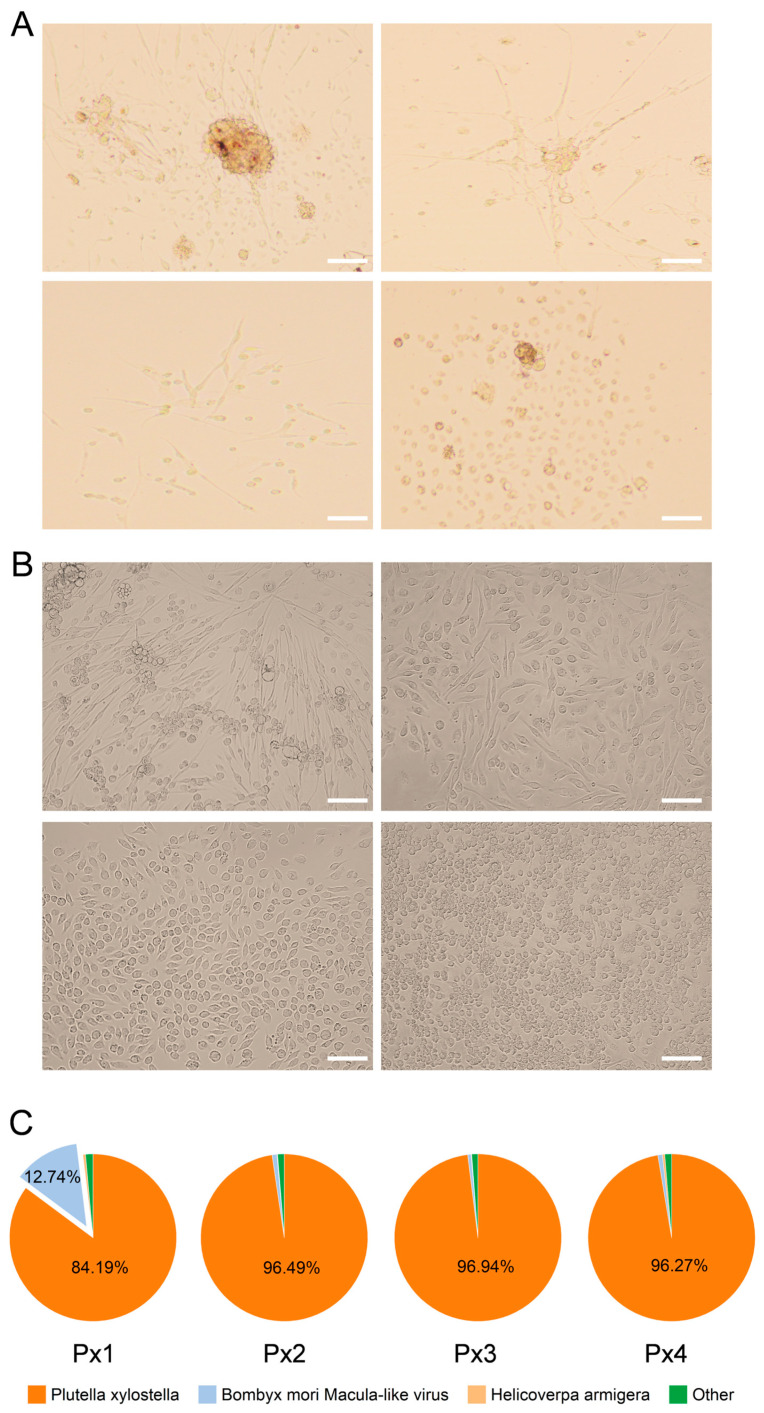
Phase contrast micrographs of cell lines and the primary culture. (**A**) Tissue pieces and cell clumps attached to the flask and cells started to migrate out of the tissue pieces. Different forms of single cells migrated from tissue pieces and cell clumps, including long spindle-shaped cells, nerve-fiber-like cells, fibrous cells, short spindle-shaped cells, and round cells. (**B**) Cell types are more homogenous throughout the culture in these sublines. Cell types mainly include fibrous, long spindle-shaped, short spindle-shaped, and round. Taken with a fluorescence microscope, the exposure time and excitation light intensity were consistent during the shooting. Scale bar: 100 μm. (**C**) The percentages of reads aligned to different species in the Px1–Px4 cell line are shown.

After the primary culturing of *P. xylostella* embryonic cells in TNM-FH medium containing 20% FBS for 4 h, small adhesion plaques appeared in the embryonic tissue. Following the replacement of the medium and the removal of non-adherent cells and cell debris, the adhesion tissue gradually increased, and new cells migrated from the tissue block. During this period, some tissues became more active and exhibited rhythmic contractions (Figure 1A and Video S1). The cell flasks predominantly displayed four morphologies: long spindle-shaped cells containing tissue blocks, cells migrating from the tissue, nerve-fiber-like cells, dispersed fibrous cells, and dispersed short spindle-shaped and round cells (Figure 1A). After 5 months of culture, four cell lines, designated Px1 to Px4, were isolated and cultured separately for passaging in TNM-FH medium containing 10% FBS. By the 20th generation, the cell growth status was robust, and the passaging process was stable, indicating that the cell lines had been successfully established. The four cell lines were sent for transcriptome sequencing, and it was found that the number of reads matching the *P. xylostella* genome in the Px2 to Px4 samples accounted for more than 96% of the total surveyed reads (Figure 1C), indicating that these cells were all derived from *P. xylostella*. In total, 12.74% of the reads in the Px1 cell line matched the *B. mori* macula-like virus. Consequently, the Px1 cell line was discarded, while the other cell lines continued to be cultured, and have been passaged more than 80 times to date. To obtain cell lines with consistent homology, four single-cell cultured lines were derived from the Px4 cell line. Their primary morphologies were fibrous, long spindle-shaped, short spindle-shaped, and round (Figure 1B), and they were designated as Px4-1, Px4-2, Px4-3, and Px4-4. Among these, Px4-2 was selected for subsequent measurements.

### 2.2. Analysis of PxEF1α Promoter Activity

**Figure 2:**
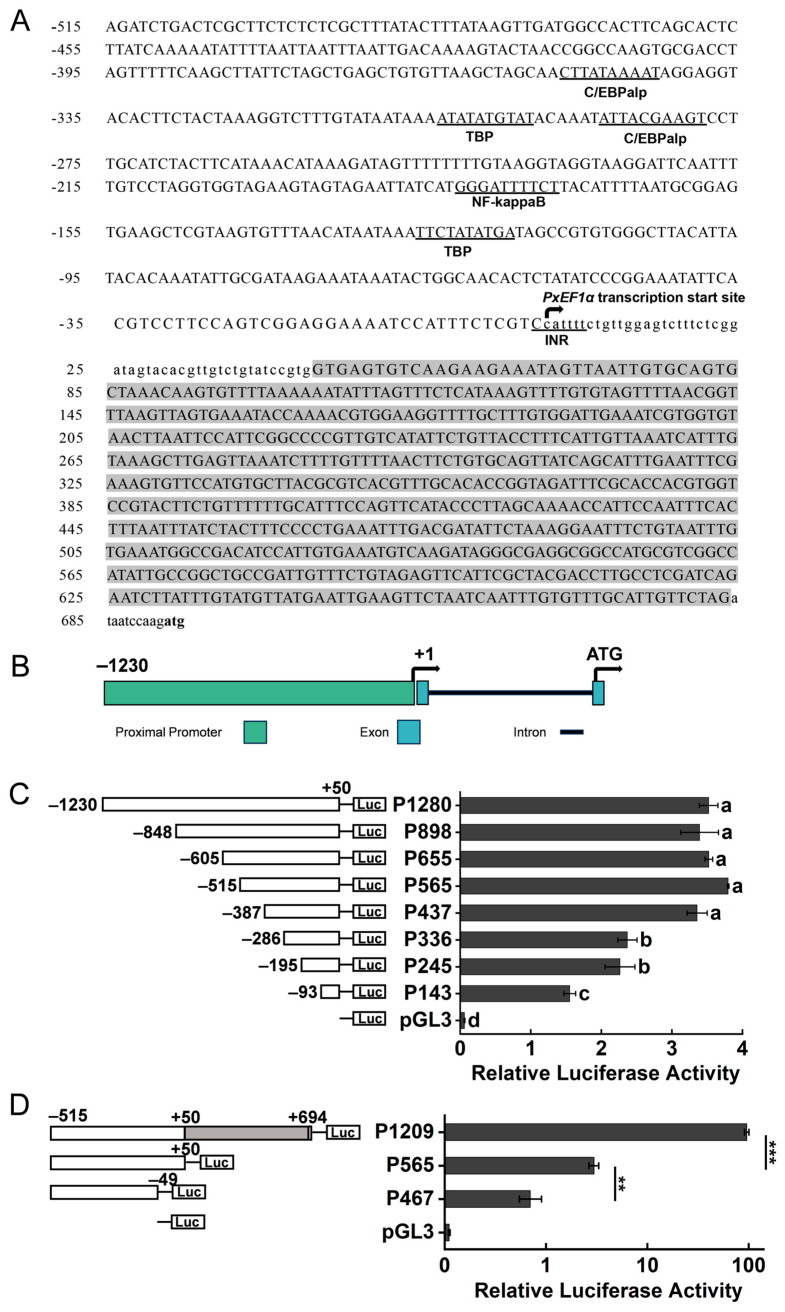
The sequence analysis of the PxEF1α promoter and transcriptional activity analysis of the candidate promoter in px4-2 cells. (**A**) The sequence analysis of the PxEF1α promoter. The grey region represents intron sequence. The arrows mark the transcription start site of PxEF1α, and the bold characters represent the translation initiation codon (ATG) of PxEF1α. The underlined sequences represent possible potential binding sites for various transcription factors. (**B**) The structure of the PxEF1α promoter. The transcription initiation site and initiation codon are represented by arrows. The green box represents the upstream region of the transcription start site, the blue box represents the exon region flanking the intron, and the black box represents the intron region. (**C**) The driving efficiency of the PxEF1α promoter after 5′ truncation. Significant differences between groups are indicated by lowercase letters (Fisher LSD, *p* < 0.05). (**D**) The driving efficiency of the PxEF1α promoter after 3′ truncation. Note: ** *p* < 0.01; *** *p* < 0.001; n = 3–4 in each group.

Obtaining highly active endogenous promoters in *P. xylostella* cells is essential for conducting multi-gene co-expression studies to further investigate the physiological regulatory processes. The transcriptome results indicated that the *elongation factor 1 alpha* (*PxEF1α*) gene exhibited the highest expression level across all samples (Table 1). The full-length 5′ untranslated region (5′ UTR) of the *PxEF1α* mRNA was determined using 5′ RLM-RACE. Subsequently, a candidate promoter sequence measuring 1924 base pairs (bp), located upstream of the PxEF1α start codon, was obtained through PCR amplification. The transcription start site (TSS) cytosine nucleotide (C) was designated as +1. The start codon is located at +695. Notably, there is an intron measuring 634 bp located between +51 and +684 (Figure 2A). The gene structure composition of the candidate PxEF1α promoter is illustrated in Figure 2B.

To assess the driving efficiency of the PxEF1α promoter, a dual-luciferase reporter assay was performed using the Px4-2 cell line. The results indicated that P565 (−515/+50) exhibited the highest driving activity in the 5′ end truncation test (Figure 2C). Furthermore, the driving activity of promoter P336 decreased by 37.64% compared to P565, which was statistically significant (*p* < 0.001). Additionally, the promoter activity of P143 decreased by 31.44% compared to P336, which was also statistically significant (*p* < 0.05). It is presumed that key motifs exist between positions −387 to −286 and −195 to −93, which have a substantial impact on promoter activity. The Alibaba 2.0 database (http://gene-regulation.com/pub/programs.html, accessed on 6 January 2025) was utilized to predict potential transcription factor binding sites for these two regions. The transcription factors C/EBPα, TBP, and NF-κB may play crucial roles in regulating the driving efficiency of PxEF1α (Figure 2A).

**Figure 3:**
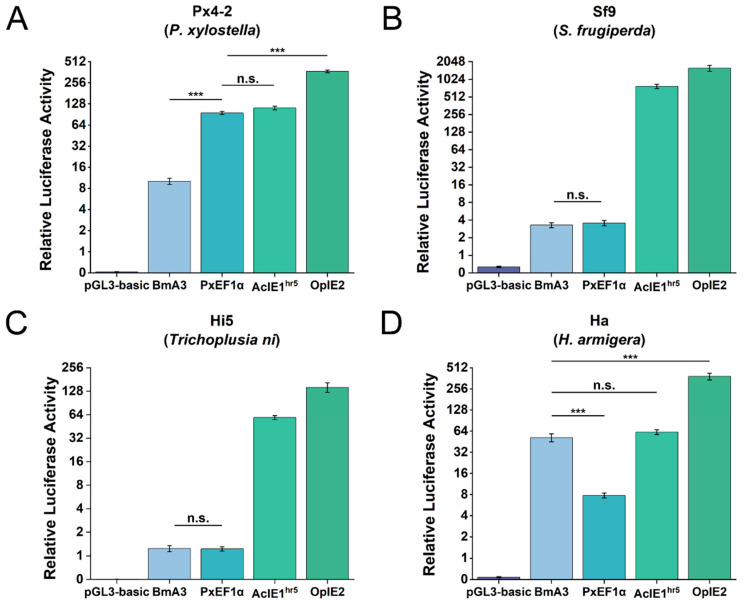
Comparison of the transcriptional activity of the PxEF1α promoter with the BmA3, AcIE1^hr5^, and OpIE2 promoters in various insect cell lines. The promoters were ligated to the luciferase reporter gene, and the plasmid vectors were transfected into different insect cell lines: Px4-2 (**A**); Sf9 (**B**); Hi5 (**C**); Ha (**D**). Forty-eight hours after transfection, the luciferase activity was measured. Note: *** *p* < 0.001; n.s., not. significant; n = 3–4 in each group.

The driving activity of the promoter P565 accounted for only 3.39% of the AcIE1^hr5^ activity (Figure 3A). It is presumed that the intron sequence downstream of the transcription start site (TSS) influences the driving activity of the PxEF1α promoter. We inserted the deleted intron sequence into the pGL3-p565 plasmid to construct the P1209 (−515/+694) test plasmid. Compared to P565, the P1209 promoter activity increased by 2418%, with a statistically significant difference (*p* < 0.001) (Figure 2D). To verify the key functions of the TSS and the upstream adjacent sequence, we constructed the p467 (−515/−49) test plasmid. Compared to P565, only about 28.37% of the activity of the P467 candidate promoter remained, and this difference was statistically significant (*p* < 0.001). In conclusion, the P1209 candidate promoter exhibited the highest activity and was designated as the PxEF1α promoter.

### 2.3. Promoters Regulate the Expression of Firefly Luciferase in Various Insect Cell Types

Using the dual-luciferase reporter assay system, we compared the PxEF1α promoter with the AcIE1^hr5^, OpIE2, and BmA3 promoters across four different insect cell lines (Figure 3 and Table S6). The results indicated that the OpIE2 promoter exhibited the highest transcriptional activity among the four cell lines, particularly in the Sf9 cell line, where the luc/rluc ratio reached 1589.61. The transcriptional activity of the AcIE1^hr5^ promoter followed closely behind that of the OpIE2 promoter across all four cell lines. The results demonstrated that the two baculovirus-derived promoters exhibited strong driving activity in various insect cell lines. In contrast, the transcriptional activity of the BmA3 promoter varied significantly across different cell lines. The BmA3 promoter only showed the transcriptional activity in the Ha cell line, with a luc/rluc ratio of 13.44% of the OpIE2 promoter, which was not significantly different from that of the AcIE1^hr5^ promoter (*p* = 0.28). The transcriptional activity of the PxEF1α promoter in different cell lines was particularly noteworthy. In the Px4-2 cell line, the PxEF1α promoter demonstrated strong transcriptional activity, with a luc/rluc ratio of 25.63% of the OpIE2 promoter, which was not significantly different from the activity of the AcIE1^hr5^ promoter (*p* = 0.12). In the Sf9, Hi5, and Ha cell lines, the PxEF1α promoter was nearly inactive, with luc/rluc ratios of less than 8. These findings reveal that insect-derived promoters, such as the PxEF1α and BmA3 promoters, exhibit varying transcriptional activities across different insect cell lines.

### 2.4. Promoters Regulate the Expression of EGFP in Various Insect Cells

**Figure 4:**
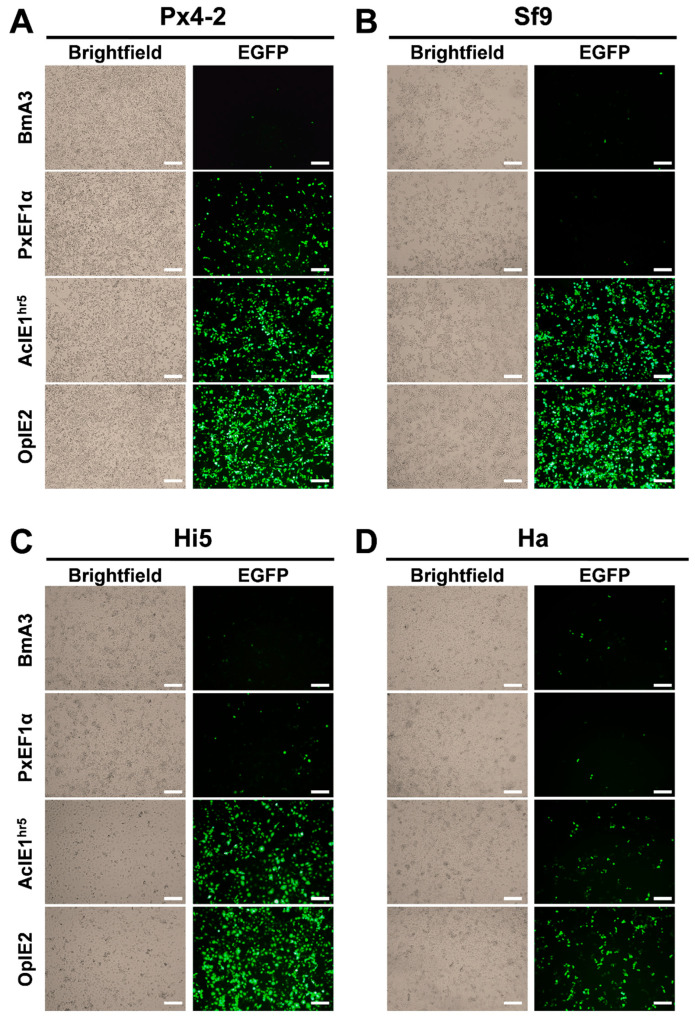
Visualization of the drive efficiency levels of the PxEF1α promoter in four different insect cell lines. Fluorescence images illustrate EGFP expression driven by the PxEF1α, BmA3, AcIE1^hr5^, and OpIE2 promoters in Px4-2 (**A**), Sf9 (**B**), Hi5 (**C**), and Ha (**D**) insect cells. An inverted fluorescence microscope was utilized to capture images, ensuring consistent exposure time and excitation light intensity throughout the imaging process. Scale bar: 200 μm.

The construction of an enhanced green fluorescent protein (EGFP) expression system driven by various promoters provides a convenient method for evaluating promoter activity. The results indicated that after transfecting four plasmids into Ha cells, the fluorescence signals were weaker compared to those in the other three cell types (Figure 4D). Notably, the order of promoter activity, from strongest to weakest, was OpIE2 > AcIE1^hr5^ > BmA3 > PxEF1α, with corresponding fluorescence intensities of 40.73 ± 1.70, 11.96 ± 2.04, 10.63 ± 1.22, and 1.73 ± 0.66, respectively. In Sf9 and Hi5 cells, strong fluorescence signals were detected for both the AcIE1^hr5^ and OpIE2 promoters, with the fluorescence intensity generated by the OpIE2 promoter being greater than that of the AcIE1^hr5^ promoter. This finding was consistent with the results of the dual-luciferase activity assay (Figure 3). The PxEF1α promoter was able to generate a strong fluorescence signal in Px4-2 cells, with a fluorescence density of 45.12 ± 0.44. Although this density was lower than that of the OpIE2 promoter, it was comparable to that of the AcIE1^hr5^ promoter (Figure 3A and Figure 4A). The relative expression levels of EGFP driven by different promoters are shown in Figure S1.

### 2.5. The PxEF1α Promoter Regulates the Expression of the Kir2A Gene in Px4-2 Cells

**Figure 5:**
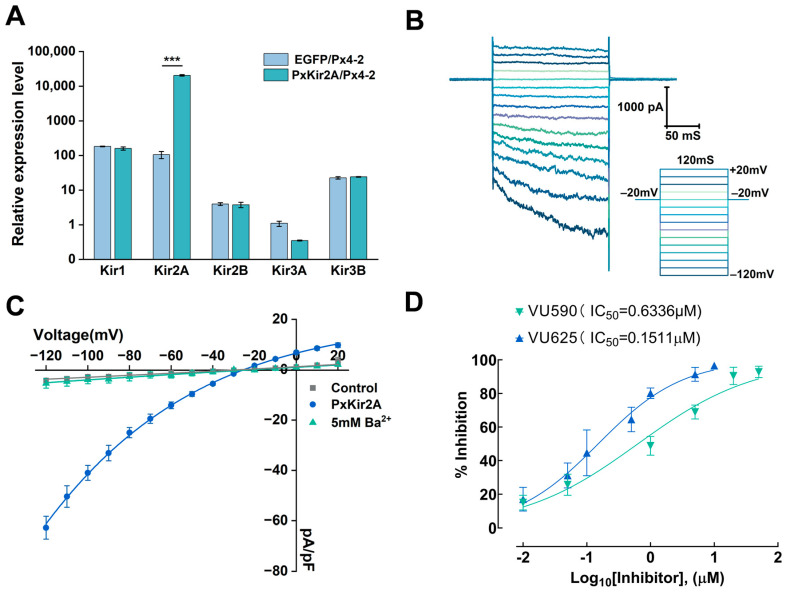
The effect of PxKir2A expression on the mRNA levels of other Kir subunits and the electrophysiological properties of PxKir2A channels in Px4-2 cells. (**A**) Relative expression of the Kir subunits in Px4-2 cells (mean ± SEM); *** *p* < 0.001; n = 3–4 in each group. (**B**) Currents recorded from Px4-2 cells expressing the PxKir2A channel. The voltage pulses range from −120 mV to +20 mV in increments of 10 mV. (**C**) The current–voltage relationships for PxKir2A are presented. The normalized current–voltage relationships for cells expressing PxKir2A, both with and without Ba^2+^, are shown (n = 4–6). (**D**) Concentration–response curves for VU590 and VU625 are illustrated. The IC50 value for VU590 was 0.6336 μM, with a 95% confidence interval (CI) range of 0.4925–0.8120 μM. The IC50 value for VU625 was 0.1511 μM, with a 95% CI range of 0.1230–0.1852 μM. These IC50 values were determined using a non-linear curve fitting analysis (log [drug] vs. normalized response, variable slope) in Prism 8 (n = 5 independent experiments).

Previous studies have demonstrated the presence of five inwardly rectifying potassium channel (Kir) subunits in *P. xylostella* [31]. In this study, a quantitative reverse transcription polymerase chain reaction (qRT-PCR) was employed to compare the expression levels of five Kir genes in PxKir2A/Px4-2 and EGFP/Px4-2 cells. The results indicated that compared to pEF1α-EGFP transfection, the expression level of the PxKir2A gene in the Px4-2 cells increased by approximately 196-fold, while the mRNA levels of PxKir1, PxKir2B, PxKir3A, and PxKir3B did not show significant differences from the control (Figure 5A). A further analysis revealed that in cells transfected with the PxKir2A expression plasmid, the mRNA level of PxKir2A was 143 ± 4.97 times that of PxKir1, 4859 ± 168.33 times that of PxKir2B, 20,580 ± 1021.52 times that of PxKir3A, and 1047 ± 36.27 times that of PxKir3B. The expression of PxKir2A was greater than the total expression of the other Kir subunits.

The whole-cell patch clamp recordings indicated that the PxKir2A channel could be activated at a membrane potential of −120 mV. During the depolarizing voltage steps, the inward current gradually decreased (Figure 5B), while no significant inward current was observed in non-transfected Px4-2 cells (Figure 5C). Additionally, we introduced Ba^2+^, a non-specific inhibitor of Kir channels, into the extracellular fluid for our analysis and found that Ba^2+^ at a final concentration of 5 mM could almost completely inhibit the current of the PxKir2A channels (Figure 5C). This finding is consistent with the previously reported electrophysiological properties of insect Kir channels.

To investigate the electrophysiological properties of PxKir2A channels, we assessed the inhibitory effects of the small molecule inhibitors VU625 and VU590 on these channels. The results demonstrated that VU625 could inhibit approximately 90% of the inward current of the PxKir2A channel, while VU590 inhibited about 70% of the current at the same concentration. The half-maximal inhibitory concentration (IC_50_) of VU625 was determined to be 0.1511 μM (R^2^ = 0.9539), whereas the IC_50_ of VU590 was found to be 0.6336 μM (R^2^ = 0.9555) (Figure 5D).

## 3. Discussion

The first cell line of *P. xylostella* was established from pupae in 1983 [32]. Since then, the *P. xylostella* cell line has been widely utilized for the amplification of insect baculoviruses [7,32] and for elucidating the molecular mechanisms of viral infection [33,34]. Compared to Sf9 and Hi5 cells, *P. xylostella* cells have fewer applications in insect physiology and molecular biology [11]. Embryonic cell lines of *P. xylostella* were established and reported in 2017 [7], and these have since been employed for gene editing [35]. In response to research needs, four monoclonal cell lines of *P. xylostella* with varying morphologies were obtained. These cell lines provide valuable research tools for studying the molecular mechanisms of insecticide resistance and for discovering new pesticide targets. Unexpectedly, the Px1 cell line exhibited a high level of *B. mori* macula-like virus (BmMLV) compared to other cell lines. BmMLV is a positive-sense, single-stranded RNA virus that was first identified from the cDNA library of the *B. mori* ovary-derived BmN-4 cell line. Almost all *B. mori*-derived cell lines are persistently infected with BmMLV, and this infection does not lead to lethality [36]. Infection studies have revealed that persistent infections with BmMLV are not established in Sf9 cells [37]. In this study, BmMLV was detected in cell lines derived from *P. xylostella* embryos. It is presumed that BmMLV can also establish persistent infections in *P. xylostella* cells, which enhances our understanding of the infection capabilities of BmMLV. The Px1 to Px4 cell lines were established based on their varying adhesion abilities to cell culture flasks. To investigate gene function in these cells, procedures such as plasmid transfection, cell fixation, imaging, and electrophysiological testing are necessary. These processes require frequent washing of the cells with different media or buffers. Selecting cell lines with strong adhesion properties can minimize cell loss during washing and increase the number of cells available for the final analysis. Consequently, in this study, the Px4 cell line and the elongated spindle-shaped cells (Px4-2) derived from it were chosen to represent *P. xylostella* cells for the promoter activity analysis and electrophysiological studies.

Promoters respond to external regulatory signals and control the expression of key genes, which is essential for the precise regulation of cellular and organismal functions [38]. However, significant differences in driving activity have been observed across various cell types [20]. This study compared the driving activities of the BmA3 and PxEF1α promoters in different cell types and found that although both are derived from lepidopteran sources, the BmA3 promoter exhibited minimal driving activity in Ha cells and almost no activity in the other three cell types. This finding sharply contrasts with previous reports regarding the activity of this promoter in silkworm cells [19,39]. Similarly, the PxEF1α promoter demonstrated higher activity in Px4-2 cells but exhibited weaker activity in other cell types. This limitation may hinder its potential application across various cell lines. However, as the research advances—such as through transposase-mediated insect transgenes or the construction of stable expression cell lines (e.g., the Tn5 transposable system)—the simultaneous expression of multiple genes is often necessary. The AcIE1^hr5^ and OpIE2 promoters may not sufficiently fulfill the requirements for the co-expression of multiple genes within the same cells. In contrast, the PxEF1α promoter can provide the necessary support. Additionally, because the endogenous promoter is regulated by the host cells, it contributes to the long-term stable expression of target genes [20]. In studies involving *Bombyx mori* and *Drosophila*, endogenous promoters are predominantly employed to drive the expression of target genes in insects [40,41]. In summary, this study presents an alternative or supplementary option for the expression of exogenous genes in *P. xylostella* cells, alongside the AcIE1^hr5^ and OpIE2 promoters.

Mammalian cells, particularly HEK293T and CHO cells, are extensively used to study ion-channel-related properties due to their high transfection efficiency [42,43]. *Xenopus* oocytes facilitate exogenous gene expression and are commonly employed in two-electrode voltage clamp technology to investigate the characteristics of insect ion channels [29]. In this study, we successfully achieved the overexpression of Kir2A in *P. xylostella* cells using the established Px4-2 cell line and the identified PxEF1α promoter. Following transfection with the pEF1α-PxKir2A vector, the expression of PxKir2A increased approximately 196-fold compared to control cells, and we successfully recorded the channel current of PxKir2A. It is hypothesized that specific cofactors are necessary for the formation of Kir2 channels in *P. xylostella* cells, and further investigation is required to understand the varying performance of Kir2A across different test systems [24]. Notably, VU625 exhibited a stronger inhibitory effect on Kir2A compared to the PxKir1 channel. This observation aligns with previous findings that VU590 demonstrates higher specificity for the Kir1 channel in mosquitoes [25]. This study paves the way for further exploration of the channel characteristics of PxKir2A and the identification of highly effective and specific inhibitors to investigate the physiological functions of Kir2A.

## 4. Materials and Methods

### 4.1. Insects, Cells, and Chemicals

*P. xylostella* was cultured as previously described by Huo et al. [44]. In this study, we established an embryonic cell line of *P. xylostella*, designated as Px4-2. The *S. frugiperda* pupal ovarian tissue cell line (Sf9) was obtained from Zhong Qiao Xin Zhou Biotechnology Company in Shanghai, China. The *T. ni* ovarian cell line (Tn5B1-4, Hi5) and the *H. armigera* embryonic cell line (QB-Ha-E5, Ha) were generously provided by Professor Huazhu Hong from Central China Normal University [45,46]. All cell lines were cultured and passaged in our laboratory. The four types of cells were maintained in a constant temperature incubator set at 28 °C. The Px4-2, Sf9, and Hi5 cells were cultured in TNM-FH insect medium supplemented with 10% fetal bovine serum (FBS), 100 U/mL penicillin, and 100 μg/mL streptomycin. The Ha cells were cultured in Sf900 II medium (Thermo Fisher Scientific, Waltham, MA, USA) containing 7% FBS.

The barium chloride (BaCl_2_, 99%, CAS: 10361-37-2) was procured from Sigma-Aldrich (St. Louis, MO, USA). The VU625 (97%, CAS: 901008-62-6) and VU590 (96.35%, CAS: 313505-85-0) were obtained from MedChemExpress (Monmouth Junction, NJ, USA). Both compounds were dissolved in anhydrous dimethyl sulfoxide (DMSO) and diluted to serial concentrations prior to their application in the cell bath solution. The final concentration of DMSO was maintained at less than 0.05% throughout the experiments. Additional chemicals and reagents utilized in this study were sourced from Vazyme (Nanjing, Jiangsu, China) and Macklin (Shanghai, China).

### 4.2. Primary Cell Culture and Cell Line Isolation

The primary cultures were initiated from one-day-old eggs of *P. xylostella*. The eggs were surface-sterilized using 10% sodium hypochlorite followed by 75% ethanol for 10 min. After two washes with sterile deionized water and two additional washes with TNM-FH medium, the eggs were crushed in TNM-FH medium supplemented with 10% FBS [7]. The resulting cell suspension was transferred into a 25 cm^2^ plastic culture flask and incubated at 28 °C. Depending on the cell growth status, the culture medium should be changed every 5 to 10 days. To minimize the impact on cell growth, one-fourth of the original culture medium should be retained during each change.

When viable cell populations began to expand significantly in the culture flasks, the cells were separated and cultured according to their adhesion properties. The cells that were gently shaken off the culture bottle were designated as Px1, while those that were normally shaken off were labeled Px2. The cells that were carefully dislodged using a pipette were classified as Px3, and those that were difficult to remove were referred to as Px4. The subculture split ratio was 1:2 during the early passages of the cell lines, gradually increasing to between 1:4 and 1:10 after 20 passages, depending on the specific cell lines, for routine weekly subcultures. Stable cell lines underwent transcriptome sequencing to confirm that all cultured cells originated from *P. xylostella.* To obtain a homogeneous cell population, single-cell cloning of the Px4 cell line was performed using the limiting dilution method for single-cell selection and culture [47]. Four distinct cell lines, Px4-1 to Px4-4, were established based on their morphological differences.

### 4.3. RNA Sequencing

To assess the PxEF1α promoter activity and confirm that the constructed cell line was derived from *P. xylostella*, we analyzed the transcriptional profile of the Px1-4 cells using RNA sequencing. Cell samples of *P. xylostella* were collected and transported on dry ice to Shanghai Ouyi Biomedical Technology Co., Ltd. (Shanghai, China) for RNA extraction, library construction, and sequencing. The sequencing strategy, acquisition of clean reads, and calculation of gene expression FPKM values using the reference genome (RefSeq accession number: GCF_000330985.1) were employed as previously described [48]. Here, 500,000 clean reads from each sample were randomly selected and compared against the nucleotide sequences (NT) database to assess potential contamination. Contamination detection was performed using BLASTn with the following parameters: e-value ≤ 1 × 10^−10^, similarity > 90%, and coverage > 80% [49].

### 4.4. 5′ RNA Ligase-Mediated Rapid Amplification of cDNA Ends (5′ RLM-RACE)

The total RNA from five one-day-old fourth-instar *P. xylostella* larvae was extracted using the Total RNA Extraction Reagent (Vazyme) according to the manufacturer’s instructions. The transcription start site of the *P. xylostella elongation factor 1 alpha* (*PxEF1α*) gene was determined using a First Choice RLM-RACE kit (Thermo Fisher Scientific, Waltham, MA, USA), following the method described by Liu et al. [50]. Briefly, the full-length 5′ untranslated region of PxEF1α mRNA was amplified using a nested PCR strategy. Two specific reverse primers, +894-R and +797-R (Table S1), were designed for the *PxEF1α* mRNA (XM_011562844). The amplification conditions included an initial denaturation step at 95 °C for 3 min, followed by 30 cycles of denaturation at 95 °C for 15 s, annealing at 57 °C for 15 s, elongation at 72 °C for 1 min, and a final extension at 72 °C for 5 min. The final product was cloned into the pCE-Zero vector and confirmed using Sanger sequencing.

### 4.5. Cloning of the Promoter

The RACE sequences were searched in the *P. xylostella* whole-genome shotgun contigs database (taxid: 51655), leading to the identification of scaffold AHIO01015874. Currently, the PxEF1α promoter has only been reported in mammals, with a length not exceeding 1300 bp [13]. In this study, specific primers, PxEF1α_pF and PxEF1α_pR, were designed using a 2 kb upstream sequence of the coding sequence (CDS) region as a reference. A PCR strategy was employed to amplify the total candidate promoter sequences, spanning from −1230 bases upstream of the transcription start site to +694 bases downstream (−1230/+694). The PCR product was purified and cloned into the pCE-Zero vector to create the pCE-P1924 recombinant plasmid. Notably, a 634 bp intron was located at the 11th base upstream of the starting codon. Given that the intron is situated near the CDS region, only the upstream sequence of the intron (P1280, −1230/+50) was amplified for the promoter activity analysis at the outset of the experiment. The primers are listed in Table S1.

### 4.6. Plasmid Construction

To identify the core promoter region, we constructed test plasmids containing various candidate promoters. The pCE-P1924 recombinant plasmid served as a template for PCR amplification to obtain truncated fragments of the different candidate promoters, which were then seamlessly cloned into the pGL3-Basic vector. Specifically, the following fragments were amplified as P1280 (−1230/+50), P898 (−848/+50), P655 (−605/+50), P565 (−515/+50), P437 (−387/+50), P336 (−286/+50), P245 (−195/+50), and P143 (−93/+50), using the specific primers listed in Table S2. The vector backbone sequences of the pGL3-Basic plasmid were amplified with the pGL3_R and pGL3_F primers. The PCR products were purified using a 1% agarose gel, and the recombinant plasmids were constructed using the Uni Seamless Cloning and Assembly Kit (TransGen Biotech, Beijing, China). The constructed products were then transformed into the *E. coli* strain DH5α, and positive clones were selected and confirmed by sequencing. The sequences from −48 to +50 in pGL3-P565 were deleted to obtain pGL3-P467 using the QuikChange II Site-Directed Mutagenesis Kit (Stratagene, La Jolla, CA, USA).

Due to the low activity of the candidate promoters obtained, an upstream sequence from the +51/+694 region of the *PxEF1α* gene was inserted into the pGL3-P565 (−515/+50) plasmid using the seamless cloning strategy described above. The pCE-P1924 recombinant plasmid served as a template, and the 50F and -1R primers were utilized to amplify the +51/+694 region, which contains an intron. The vector backbone sequence of the pGL3-P565 plasmid was amplified using the pGL3-F and 50-R primers. The constructed plasmid exhibited the highest driving activity, and the sequence from −515 to +694 was designated as the PxEF1α promoter.

GFP expression plasmids containing various promoters were constructed based on the pEGFP-N1 plasmid. The OpIE2 promoter was amplified from the pIZTV5-His plasmid using the primers p-OpIE2-F and p-OpIE2-R, while the PxEF1α promoter was amplified from the pCE-1924 vector with the primers p-PxEF1α-F and p-PxEF1α-R. The AcIE1^hr5^ (GenBank: AF434923.1) and BmA3 promoters (LOC: NC_085123.1) were artificially synthesized. The vector backbone sequence of the pEGFP-N1 plasmid, excluding the CMV promoter, was amplified using the primers pEGFP-F and pEGFP-R. Four plasmids (pEF1α-EGFP-N1, pIE2-EGFP-N1, pIE1-EGFP-N1, and pBmA3-EGFP-N1) were constructed using the seamless cloning strategy described above.

The OpIE2, AcIE1^hr5^, and BmA3 promoters were amplified from the pIE2-EGFP-N1, pIE1-EGFP-N1, and pBmA3-EGFP-N1 plasmids using the Promoter_F and Promoter_R primers, respectively. The vector backbone sequence of the pGL3-Basic plasmid was amplified with the pGL3_F and pGL3_R primers. These seamless cloning strategies were employed to construct the pGL3-OpIE2, pGL3-AcIE1^hr5^, and pGL3-BmA3 plasmids. The primers mentioned above are listed in Tables S2 and S3.

### 4.7. Cell Transfection and Photography

Insect cells were cultured in 35 mm dishes at a density of 0.8 × 10^6^ cells per dish at 27 °C for a duration of 12 to 24 h. During the exponential growth phase, 2 μg of plasmid DNA per well was used to transfect the cells with FuGENE^®^ HD Transfection Reagent (Promega, Madison, WI, USA), in accordance with the manufacturer’s instructions. Four types of EGFP expression plasmids (pEF1α-EGFP-N1, pIE2-EGFP-N1, pIE1-EGFP-N1, and pBmA3-EGFP-N1), each containing different promoters, were employed to assess the activity of various promoters in insect cells and for fluorescence imaging. The fluorescence grayscale values of the images were analyzed using ImageJ software (https://imagej.net/ij/ accessed on 15 January 2025). Additionally, the co-transfection of 2 μg of pEF1α-PxKir2A and 0.5 μg of pIE1-EGFP-N1 was conducted in Px4-2 cells for electrophysiological assays.

### 4.8. Dual-Luciferase Activity Assay

The insect cells were cultured in 24-well plates (Nest, Wuxi, Jiangsu, China) at a density of 1.5 × 10^5^ cells per well prior to transfection. A total of 2.5 ng of pRL-OpIE2 and 0.6 μg of a recombinant plasmid containing the full-length PxEF1α promoter or its truncated sequences were combined for transfection into the cells in each well [50]. At 48 h post-transfection, the cells were harvested to measure the firefly and *Renilla* luciferase activities using the Dual-Luciferase^®^ Reporter Assay Kit (Promega, Madison, WI, USA) with a Synergy 2 multi-mode microplate reader (BioTek, Winooski, VT, USA), following the manufacturers’ instructions. All reporter assays were performed in triplicate (n = 3), and the expression of the reporter genes is reported as the mean of the three experiments ± SEM.

### 4.9. Electrophysiological Assays

The full-length open reading frame (ORF) sequence of PxKir2A was obtained through a polymerase chain reaction (PCR) using specific primers and subsequently cloned into the pEASY^®^-Report Blunt Zero Cloning Vector [31]. In this study, the complete sequence of PxKir2A was amplified with Phanta Max Super Fidelity DNA polymerase using the specific primers OpIE_2F and OpIE_2R. The vector backbone sequences of the pEF1α-EGFP-N1 plasmid were generated using the specific primers EF1α_ΔEGFP-K2F and EF1α_ΔEGFP-K2R. After the PxKir2A fragment and the pEF1α-EGFP-N1 vector backbone were separated and purified using 0.7% agarose gel electrophoresis, the recombinant plasmid pEF1α-PxKir2A was constructed using the Ready-to-Use Seamless Cloning Kit. The primers mentioned above are listed in Table S4.

Single-cell patch clamp and whole-cell recording modes were employed to investigate the electrophysiological properties of the PxKir2A channels. Patch electrodes were prepared, and whole-cell patch clamp recordings were performed as previously described [44]. The electrodes were filled with an intracellular solution (in mM) of 135 KCl, 2 MgCl_2_, 1 EGTA, 10 HEPES, and 2 Na_2_ATP, at pH 6.4, adjusted with KOH, resulting in an osmotic pressure of 370 mOsm/kg, adjusted with sucrose. The bath solution contained (in mM) 90 NaCl, 50 KCl, 2 CaCl_2_, 1 MgCl_2_, 5 glucose, and 10 HEPES, at pH 6.4, adjusted with NaOH, also with an osmotic pressure of 370 mOsm/kg, adjusted with sucrose [27]. The cells were maintained at a holding potential of −20 mV and were stepped from −120 mV to +20 mV in 10 mV increments over a total duration of 120 ms. The recordings were sampled at 10 kHz and underwent series resistance compensation and low-pass filtering at 2 kHz. All recordings were conducted at room temperature (25 °C). After obtaining stable whole-cell currents, Ba^2+^, VU625, and VU590 were applied to characterize the electrophysiological properties of PxKir2A. Clampfit 10.6 software (Axon Instruments Inc., San Jose, CA, USA) was used for the data collection and analysis.

### 4.10. Quantitative Real-Time PCR

The Px4-2 cells were transfected with pEF1α-PxKir2A and pEF1α-EGFP plasmids in a 24-well plate. After culturing at 27 °C for 48 h, two wells of cells were collected for each sample. The RNA extraction, cDNA synthesis, and quantitative real-time PCR analysis were performed as previously described by Lai et al. [31]. The results from three independent samples were analyzed, and the expression differences between the various samples were compared using the 2^−ΔΔCT^ method. The primers used are shown in Table S5.

### 4.11. Statistical Analysis

The data were plotted as means ± the standard error, and curve fitting was performed using Origin 2025 (Origin Lab, Northampton, MA, USA). Significant differences resulting from experimental treatments were assessed using a Student’s *t*-test or a one-way ANOVA, followed by a Fisher’s least significant difference (LSD) post hoc test, as indicated in the figure captions. Statistical significance was determined based on a *p*-value of less than 0.05.

## Figures and Tables

**Table 1:** Genes with high-level expression in *Plutella xylostella* cell lines.

Gene_ID	Description	Px2	Px3	Px4	AVE. (fpkm)
LOC105391374	elongation factor 1-alpha	6779.87	6455.37	6476.86	6570.70
LOC105383306	heat shock 70 kDa protein cognate 4	6083.09	3606.12	3578.88	4422.70
LOC105380966	tubulin beta-1 chain	4481.64	3106.23	4111.85	3899.91
LOC105395368	40S ribosomal protein S26	3673.93	3278.9	4185.09	3712.64
LOC105387458	60S ribosomal protein L32	3927.65	3106.78	3704	3579.48
LOC105398657	40S ribosomal protein S25	3459.16	1732.38	4772.07	3321.20
LOC105393825	60S ribosomal protein L13	3301.49	2304.95	3595.51	3067.32
LOC105397135	60S ribosomal protein L40	2758.34	2063.79	3681.04	2834.39
LOC105386998	60S ribosomal protein L30	4097.38	2158.8	2229.32	2828.50
LOC105392651	60S ribosomal protein L18	3183.03	2915.76	1925.27	2674.69
LOC105386163	40S ribosomal protein S10	2881.14	2508.21	2434.86	2608.07
LOC105398808	ADP, ATP carrier protein 2	2243.29	2849.8	2616.7	2569.93
LOC105391828	60S ribosomal protein L23	3002.74	2520.08	2024.39	2515.74
LOC105382338	60S ribosomal protein L24	3021.33	1746.83	2401.96	2390.04
LOC105387239	60S ribosomal protein L35	4011.06	1746.09	1404.56	2387.24

The read counts of these genes and the corresponding calculated fpkm scores confirmed that all 15 genes were highly expressed in Px4-2 cells in our current study.

## Data Availability

All data generated or analyzed during this study are included in this published article (and its Supplementary Materials files).

## Supplementary Materials

The supporting information can be downloaded at: https://www.mdpi.com/article/10.3390/ijms26073042/s1.
