# Supplementary material for: The Elongation Factor 1 Alpha Promoter Drives the Functional Expression of Kir2A in Plutella xylostella Cells
Source: Int J Mol Sci. 2025 Mar 26;26(7):3042. doi: 10.3390/ijms26073042 (PMC11989005; doi:10.3390/ijms26073042)
Supplement: Supplementary file 1 [file ijms-26-03042-s001.zip › Supplementary materials.pdf]

Table S1. Primers for amplification of potential promoter of endogenous genes in *P. xylostella*.

Primer	Sequence (5'→3')
PxEF1α_pF	GAAATAAACTTTAATGGTGT
PxEF1α_pR	CTTGGATTATCTAGAACAA
5' RACE Outer Primer	GCTGATGGCGATGAATGAACACTG
+894-R	CGCTCAGCCTTCAGCTTGTCC
5' RACE Inner Primer	CGCGGATCCGAACACTGCGTTTGCTGGCTTTGATG
+797-R	CGATACCGCCGCATTTGTAGAT

Table S2. Primers for construction of pGL3 plasmid with different promoters.

Truncated promoter	Primer (5'→3')
P1280_F	ATTCCGGTACTGTTGAAATAAACTTTAATGGTGT
P1280_R	TCTTCCATGGTGGCTCACGGATACAGACAACGTGTACT
P898_F	ATTCCGGTACTGTTGACGCCATTGAAATGTTATTTAG
P1280_R	TCTTCCATGGTGGCTCACGGATACAGACAACGTGTACT
P655_F	ATTCCGGTACTGTTGGGTACCTAAGTATTAAAAATACATATTT
P1280_R	TCTTCCATGGTGGCTCACGGATACAGACAACGTGTACT
P565_F	ATTCCGGTACTGTTGAGATCTGACTCGCTTCTCTCT
P1280_R	TCTTCCATGGTGGCTCACGGATACAGACAACGTGTACT
P437_F	ATTCCGGTACTGTTGAAGCTTATTCTAGCTGAGCTGT
P1280_R	TCTTCCATGGTGGCTCACGGATACAGACAACGTGTACT
P336_F	ATTCCGGTACTGTTGTACGAAGTCCTTGCATCTACTTCATA
P1280_R	TCTTCCATGGTGGCTCACGGATACAGACAACGTGTACT
P245_F	ATTCCGGTACTGTTGGTAGAATTATCATGGGATTTTCTTAC
P1280_R	TCTTCCATGGTGGCTCACGGATACAGACAACGTGTACT
P143_F	ATTCCGGTACTGTTGGTAGAATTATCATGGGATTTTCTTAC
P1280_R	TCTTCCATGGTGGCTCACGGATACAGACAACGTGTACT
Promoter_F	CATTCCGGTACTGTTGGTGGATAACCGTATTAC
Promoter_R	TCTTCCATGGTGGCTGAGCTCGAGATCTGAGT
pGL3_F	AGCCACCATGGAAGACGC
pGL3_R	CAACAGTACCGGAATGCC
P467-R	GATATAGAGTGTTGCCAGTATTTA
pGL3_F	AGCCACCATGGAAGACGC
50-F	TTGTCTGTATCCGTGAGTACACGTTGTCTGTATCCGTG
-1-R	TCTTCCATGGTGGCTCTTGGATTATCTAGAACAA

pGL3_F	AGCCACCATGGAAGACGC
50R	CACGGATACAGACAACGTGTACT

The underlined part in the table indicates the homology arm added to the primer.

Table S3. Primers for construction of p-EGFP plasmid with different promoters.

Primer Name	Sequence (5'→3')
pEGFP-F	GGTTTAGTGAACCGTCAGATCC
pEGFP-R	CGGAACTCCATATATGGGCTAT
p-OpIE2-F	ATATATGGAGTTCCGCATGATGATAAACAATGTATGGTGCT
p-OpIE2-R	ACGGTTCACTAAACCAACAGATGCTGTTCAACTGTG
p-AcIE1-F	ATATATGGAGTTCCGCGCGTAAAACACAATCAAGTACG
p-AcIE1-R	ACGGTTCACTAAACCATCTCTTGTCGCCGCCAG
p-PxEF1α_F	ATATATGGAGTTCCGGAAATAAACTTTAATGGTGT
p- PxEF1α-R	ACGGTTCACTAAACCTCTTGGATTATCTAGAACAA
p-BmA3P-F	ATATATGGAGTTCCGGGAGTCGGGGAGAGGTTACA
p-BmA3P-R	ACGGTTCACTAAACCTTGAATTAGTCTGCAAG

The underlined part in the table indicates the homology arm added to the primer.

Table S4. Primers for construction of pEF1a-PxKir2A plasmid.

Primer Name	Sequence (5'→3')
OpIE_2F	CGCAACGATCTGGTAAACAC
OpIE_2R	GACAATACAAACTAAGATTTAGTCAG
EF1α_△EGFP-K2_F	TTAGTTTGTATTGTCTGAATGCAATTGTTGTTGTTAA
EF1α_△EGFP-K2_R	TACCAGATCGTTGCGTGAGCTCGAGATCTGAGTCCG

The underlined part in the table indicates the homology arm added to the primer.

Table S5. Primers for RT-qPCR.

Primer Name	Sequence (5'→3')
PxKir1-qF	GAGGTGCTGCCGTTCTAC
PxKir1-qR	TCGACTCGATCACTCCCT
PxKir2-qF	ATTGGACAGTTCACGATACATAGGAA
PxKir2-qR	CCGAGCCAGGAGAGGATGAAG
PxKir3a-qF	GGGGGTACCGCTTCAAGAATGTTAT

PxKir3a-qR	GGAGGAACCCTGTGTGACTGAG
PxKir3b-qF	ACAAGGCTGGCTATGGCGACTA
PxKir3b-qR	GATGTACCAGAAACCAGCGAAC
PxKir4-qF	TATCATCCAGGAAGTGGCTAAGGCT
PxKir4-qR	GTCGGGAAACGCATGGTACTTGTGT
EF1-qF	GCCTCCCTACAGCGAATC
EF1-qR	CCTTGAACCAGGGCATCT

The primers in this table are cited from Lai et al. (2020).

Video S1. During primary culture, some tissues showed rhythmic contraction.

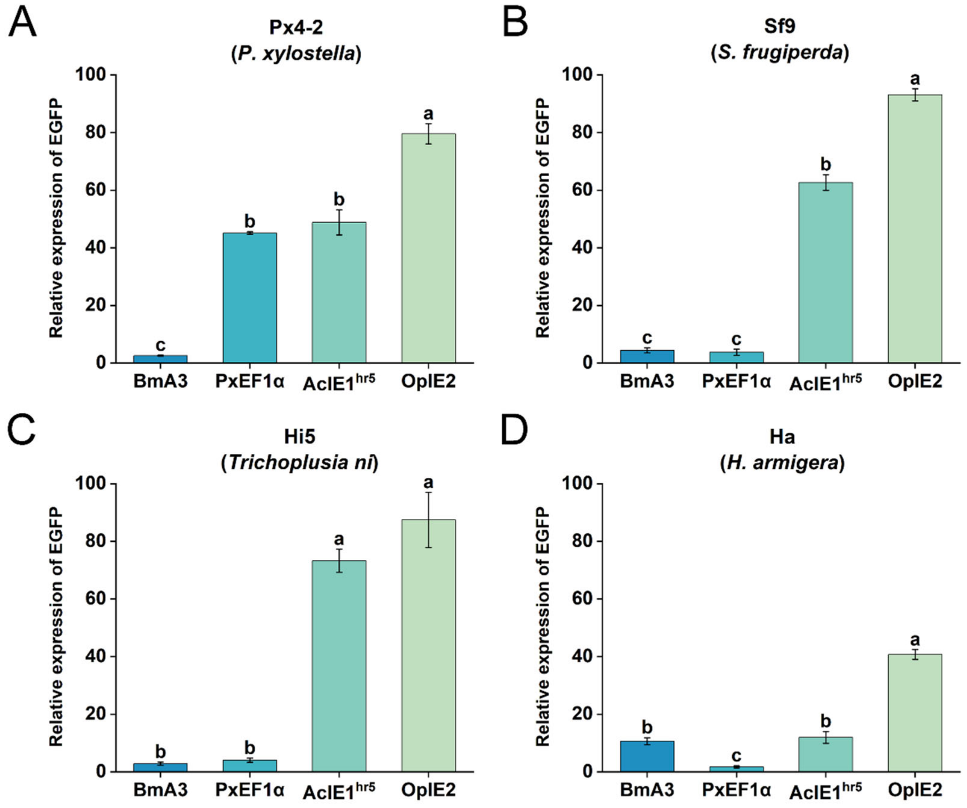

E

Cell	Promoter	Mean	SE of Mean
Px4-2	BmA3	2.58	0.19
	PxEF1α	45.12	0.44
	AcIE1^{hr5}	48.85	4.36
	OpIE2	79.56	3.52
Sf9	BmA3	4.40	0.89
	PxEF1α	3.74	1.10
	AcIE1^{hr5}	62.65	2.73
	OpIE2	93.13	2.13
Hi5	BmA3	2.84	0.57
	PxEF1α	4.03	0.71
	AcIE1^{hr5}	73.31	4.02
	OpIE2	87.46	9.57
Ha	BmA3	10.63	1.22
	PxEF1α	1.73	0.66
	AcIE1^{hr5}	11.96	2.04
	OpIE2	40.73	1.71

Figure S1. Relative expression of EGFP driven by promoters in different cell lines: Px4-2 (A), Sf9 (B), Hi5 (C), and Ha (D). The significant differences between groups are indicated by lowercase letters (Fisher LSD, $P < 0.05$). (E) Fluorescence density values of EGFP in cells driven by different promoters. SE indicates standard error.

Table S6. Dual-luciferase assay of the promoter in different cell lines

Cell	Promoter	Mean（luc/rluc）	SE of Mean
Px4-2	pGL3	0.04	0.01
	BmA3	10.14	1.05
	PxEF1α	95.63	4.87
	$AcIE1^{hr5}$	112.33	6.69
	OpIE2	373.15	16.40
Sf9	pGL3	0.34	0.04
	BmA3	3.27	0.31
	PxEF1α	3.58	0.37
	$AcIE1^{hr5}$	775.43	64.18
	OpIE2	1589.61	181.96
Hi5	pGL3	0.01	0.00
	BmA3	1.24	0.11
	PxEF1α	1.22	0.08
	$AcIE1^{hr5}$	58.76	3.36
	OpIE2	143.67	19.90
Ha	pGL3	0.11	0.03
	BmA3	51.81	6.68
	PxEF1α	7.77	0.63
	$AcIE1^{hr5}$	62.14	4.78
	OpIE2	385.51	42.36
